# Nano Eco-Scale: A scoring system for the assessment of the greenness and safety of manufactured nanomaterials for analytical and environmental applications

**DOI:** 10.1038/s41598-026-62228-5

**Published:** 2026-07-21

**Authors:** Aya A. Abdella

**Affiliations:** Department of Pharmaceutical Analytical Chemistry, Faculty of Pharmacy, Tanta University, Elgeish Street, The Medical Campus of Tanta University, Tanta, 31111 Egypt

**Keywords:** Nanotoxicity, Greenness, Eco-toxicity, Green analytical chemistry, Eco-scale score, Nanomaterials, Chemistry, Environmental sciences, Materials science, Nanoscience and technology

## Abstract

Manufactured nanomaterials (MNMs) have transformed analytical and environmental applications by improving efficiency and reducing resource use. However, the "nano-paradox" has raised concerns about the use and development of nanotechnology, complicating regulatory agencies’ ability to monitor MNM’s environmental impact. In this work, a novel metric tool, the Nano Eco-Scale, is proposed to assess the greenness and environmental safety of nanomaterials used in analytical and environmental applications. The framework is based on the principles of green analytical chemistry and is aligned with REACH regulations for nanomaterials, while incorporating recommendations from the Organization for Economic Co-operation and Development for standardized characterization and safety evaluation. The tool assigns penalty points to deviations from the ideal nanomaterial across four main domains: synthesis conditions, nanomaterial toxicity, exposure during use, and waste generation and disposal. The overall score is calculated as 100 minus the total penalty points. The Nano Eco-Scale score supports the evaluation and comparison of environmental impacts associated with MNM-based analytical applications such as sensing, environmental monitoring, and water treatment. This approach encourages detailed characterization, highlights potential ecotoxicity risks, and promotes safer-by-design strategies. As a screening tool, it enables rapid comparisons and helps guide the selection of safer and more sustainable options. Additionally, it facilitates systematic data collection that could support future classification approaches. The developed tool supports regulatory efforts and advances the responsible use of nanotechnology in analytical and environmental fields.

**Supplementary Information:**

The online version contains supplementary material available at 10.1038/s41598-026-62228-5.

## Introduction

Manufactured nanomaterials (MNMs) are engineered materials at the nanoscale, typically ranging from 1 to 100 nm, where matter exhibits unique electrical, mechanical, and chemical properties^1^. Their development has revolutionized modern science and technology by allowing control at the atomic level. Different classes of nanomaterials are reported in literature, including polymeric and drug nanoparticles, carbon-based nanomaterials (CBNMs), silica nanoparticles, and metal nanoparticles. Examples include carbon nanotubes, graphene, and quantum dots, which have significantly impacted fields such as electronics, medicine, energy, and analytical applications. Ongoing advances in synthesis methods now enable the production of MNMs with precise control over size, shape, and composition.

As nanotechnology advances, concerns about the potential toxicological effects of these materials are increasing^2^. Therefore, the Nanoscale Materials Stewardship Program (NMSP) encourages considering green chemistry and safer-by-design principles for MNMs preparation. Safety-by-design is an approach that aims to reduce hazards during material development. It works early in synthesis planning, focusing on prevention instead of correction^3^. Unlike safety-by-design, green chemistry focuses on sustainability to reduce environmental impact across the lifecycle.

**Fig. 1:**
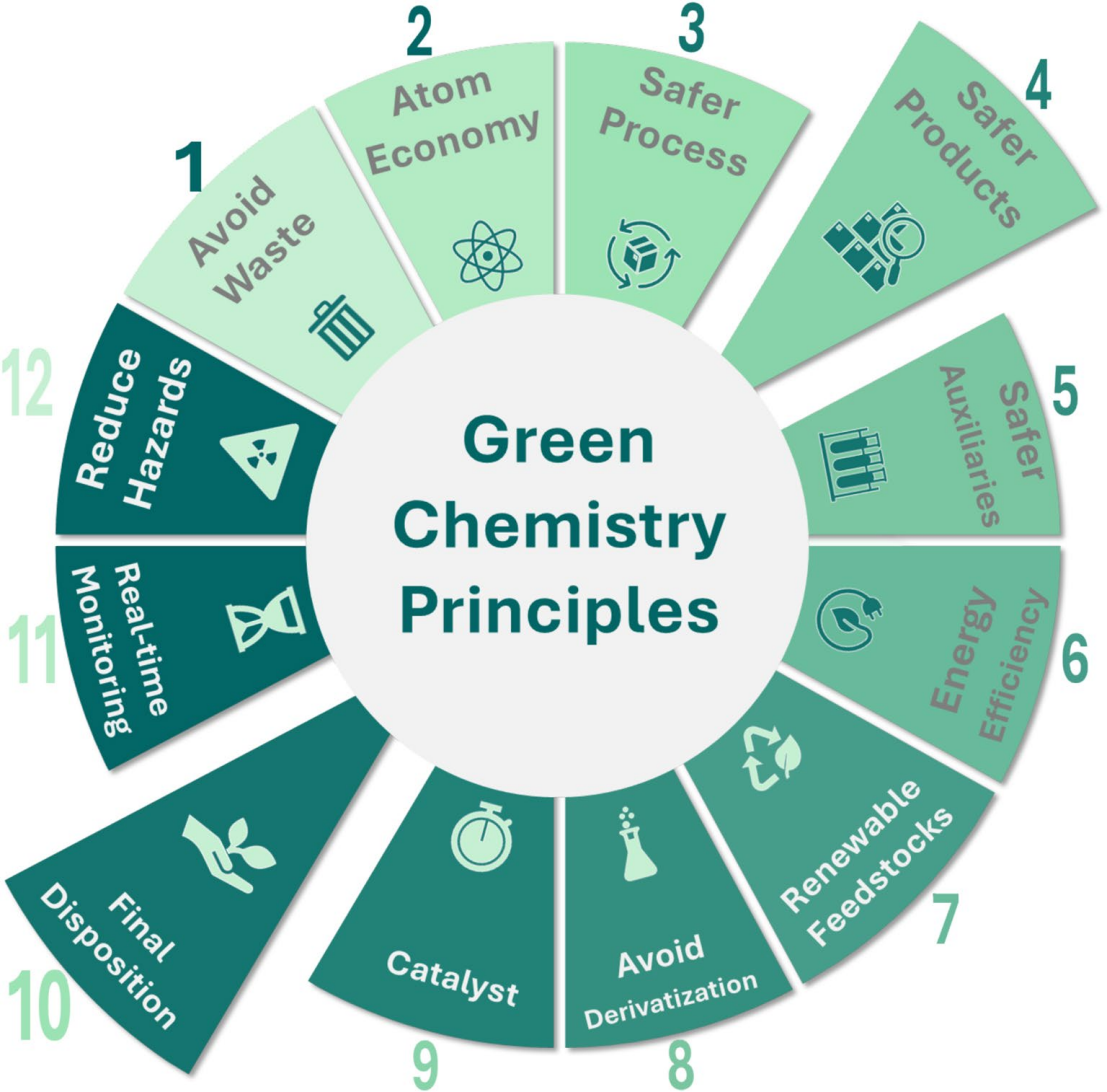
Principles of green chemistry, highlighting the remaining gaps in assessing the greenness of nanomaterials, including product safety and final disposal.

The concept of green chemistry emerged in the early 1990s as a framework for sustainable development, defined by the twelve principles introduced by Paul Anastas and John Warner, aiming to reduce or eliminate hazardous substance use and generation^4–6^ (Fig. 1). This concept later extended to analytical sciences through green analytical chemistry, which adapts these principles to evaluate and minimize the environmental impact of analytical methods^7,8^. The fast-ongoing development in this field has provided the scientific community with valuable tools to thoroughly assess and compare the ecological impact of synthetic processes and analytical methods, aiming to reduce hazard and environmental burdens. The Eco-Scale was first introduced by Van Aken to evaluate the environmental impact of organic synthesis using criteria such as yield, cost, safety, and waste^9^. This original framework focuses on chemical production and assigns penalty points based on deviations from ideal green conditions. Later, Gałuszka adapted the concept for analytical chemistry, emphasizing factors like reagent toxicity, energy consumption, and occupational risk^10^. Neither framework fully captures the unique environmental and safety considerations associated with nanomaterials, including nanoparticle synthesis, potential toxicity, and lifecycle impact (Fig. 1). Also, conventional green chemistry tools, such as E-factor and Reaction Mass Efficiency, originally designed for organic syntheses, typically do not align with the evaluation of nanomaterials.

MNMs could improve the greenness of analytical methods by reducing reagent use, lowering detection limits, and enabling faster, energy-efficient analyses^11^. However, concerns about nanomaterial toxicity, including potential environmental persistence and biological harm, require careful assessment to ensure these methods remain truly sustainable.

Over the last decade, different greenness metric tools have been applied to assess the environmental impact of MNM-based analytical methods. Current greenness metric tools in literature primarily focus on improving the greenness of the synthesis process and bulk chemicals while neglecting nano-specific properties of the product. Consequently, this underestimation of the effects of nanoscale dimensions and large surface areas leads to misinterpretation of nanomaterials’ greenness. The very small particle size can result in significant health and environmental consequences^3^. This is largely due to their high penetration ability, which allows them to transverse from the cell membrane to the nucleus, potentially damaging genetic material. Additionally, some nanomaterials are highly reactive, forming reactive oxygen species (ROS) that contribute to increased oxidative stress in cells^3^. Therefore, there is an urgent need to develop a specific tool that can effectively assess and evaluate the alignment of the MNMs to the green chemistry principles. To address this, the Nano Eco-Scale was proposed as a tailored extension that integrates principles from both systems while accounting for nano-specific risks and behaviors.

In this article, a new greenness metric tool, the Nano Eco-Scale score, was developed for the assessment of environmental impact of MNM for analytical and environmental applications. Penalty points (PPs) assigned for different parameters covering synthesis, nanomaterial properties, use, and waste disposal. The new tool is based on reporting a total score of 100-PP. To the best of our knowledge, this is the first greenness metric tool dedicated to evaluating the greenness and environmental safety of MNM-based analytical methods. Thus, the proposed Nano Eco-Scale can be a good tool supporting the safe laboratory practice of MNMs, achieving the goals of safer-by-design approaches, enabling comparative greenness evaluation, and encouraging thorough characterization and standardized toxicity testing. The tool is intended for comparative screening and early-stage assessment rather than predictive toxicity modelling or regulatory decision-making.

## Green chemistry in MNMs synthesis

MNMs pose environmental threats due to their persistence, mobility, and toxicity in air, soil, and water. They can penetrate living cells, generate reactive oxygen species, and damage DNA, proteins, and cell membranes^3^. Upon prolonged exposure, MNMs can cause inflammation, oxidative stress, and bioaccumulation in organisms. Applying green chemistry principles to MNMs (using renewable feedstocks, safer materials, and energy-efficient synthesis) prevents waste and pollution, reducing their toxic effects. Also, designing biodegradable nanomaterials ensures safe degradation after use^3^. Green chemistry turns MNM production into a cleaner and safer process aligned with environmental protection and sustainability goals^12^.

**Fig. 2:**
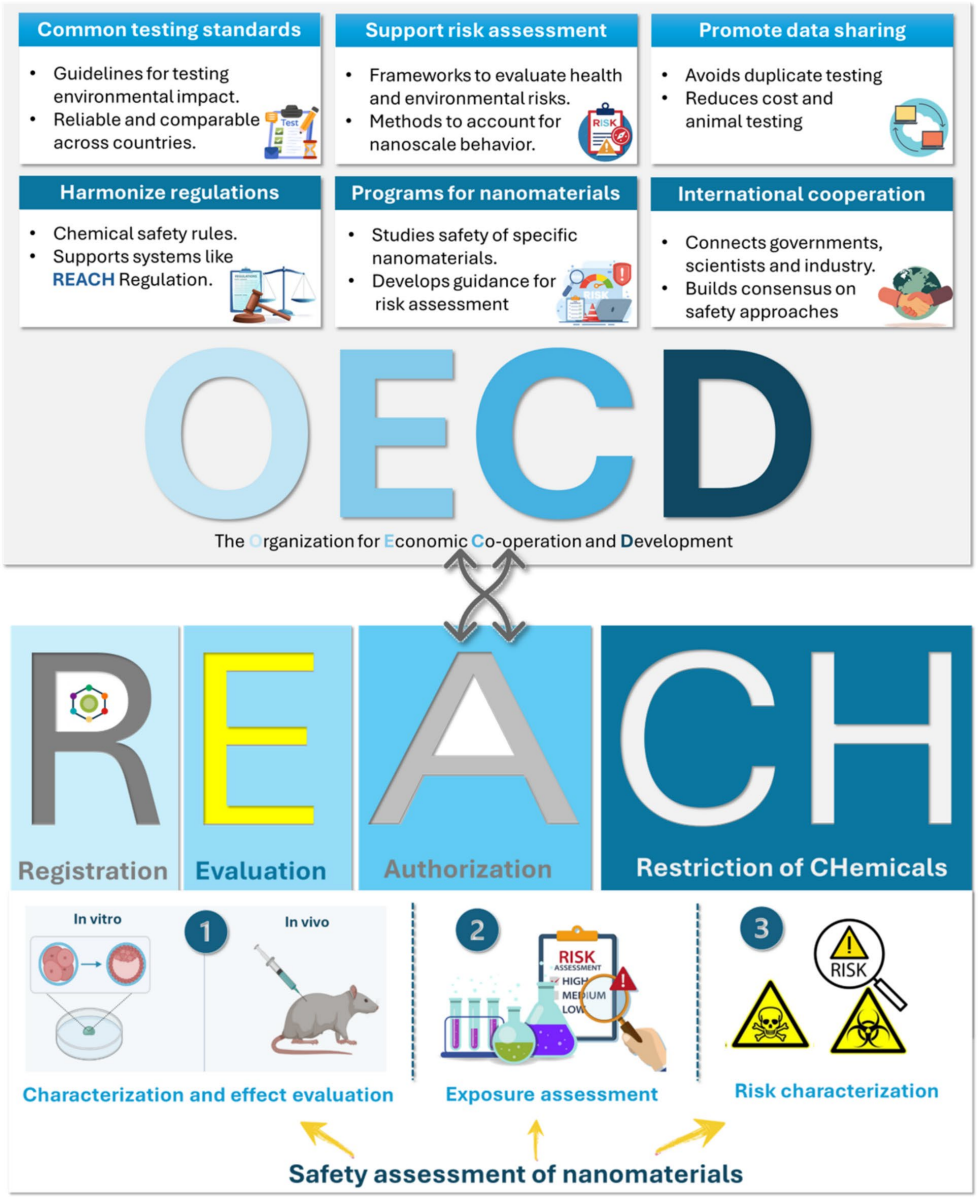
Integrated roles of REACH Regulation and Organization for Economic Cooperation and Development (OECD) in the regulatory assessment and risk management of nanomaterials.

The REACH Regulation of the European Union applies specific principles to MNMs based on their unique properties at the nanoscale. A key principle is that each nanoform of a substance requires its own risk assessment, since properties can differ significantly from the bulk form. REACH emphasizes that risk depends on the form of the material, not just its chemical identity, making the detailed characterization and nano-specific testing essential^3,13^. Also, the Organization for Economic Co-operation and Development (OECD) supports the safe use of MNMs by developing standardized test guidelines and risk assessment approaches adapted to nanoscale properties^14^. It runs targeted programs that study how nanomaterials behave in biological and environmental systems, and it promotes data sharing among countries to reduce duplicate testing and cost. The OECD also works to harmonize regulations across regions, helping ensure that safety data for nanomaterials is consistent, reliable, and accepted internationally (Fig. 2).

### Synthesis procedures

The process of MNM preparation can threaten the eco-system in different ways: waste generation, energy consumption, using hazardous reagents, and vapor/gas emissions. The selection of the synthetic approach plays an important role in shaping the properties of the formed MNMs. Top-down and bottom-up are two methodologies for approaching nanoscale. The top-down strategy entails minimizing the dimensions of the structure to the nanoscale using costly instrumentations that consume relatively large amounts of energy. However, it manages to produce nanomaterials of high purity and reproducibility. On the other hand, the bottom-up approach is mainly based on the assembly of small molecules or atoms into nanostructures through some physical or chemical processes, e.g., pyrolysis, hydrothermal autoclaving, and microwave-assisted pyrolysis^15^. This approach is considered simple, mild, and versatile but produces large amounts of impurities.

The precursors from which the MNM is synthesized could also constitute an environmental threat. Organic compounds may possess carcinogenic, mutagenic, teratogenic, corrosive, highly combustible, or explosive characteristics, among others. Furthermore, the risk may intensify over time, as evidenced by the photooxidation of ether yielding explosive peroxides^9^. It is essential to recognize that the combination of certain compounds can lead to hazardous situations (e.g., exothermic reactions between acids and bases). Accordingly, the preparation of MNMs from natural precursors could reduce the risk of hazards compared to those prepared from chemical precursors^16–20^. Also, using a highly flammable or toxic solvent can increase the risks associated with the preparation process^17^.

Regarding energy consumption, the different synthetic procedures use versatile instruments for variable durations. Accordingly, comparing the power supply of instruments per hour ignores the effect of time, leading to lack of differentiation between a method using the device for minutes and another one that uses it for hours. Therefore, the calculation of CO_2_ emissions can be a better measure for energy consumption as it takes into consideration both device energy consumption per hour and time of use**.** CO_2_ emission is calculated using: Instrument Power (KW) X Analysis time (h) X Emission factor for electricity (0.0247 kg CO2/kWh)^21^. The power of different instrumentations employed in MNM preparation for the calculation of carbon footprint is presented in Table S1.

Finally, the evolution of gases and vapors during the preparation process is considered an occupational hazard as well as an environmental threat that needs to be avoided. For this reason, using a closed system, e.g., hydrothermal autoclave, reduces the gas emissions during carbonization^22^.

### Toxicity of nanomaterials

**Fig. 3:**
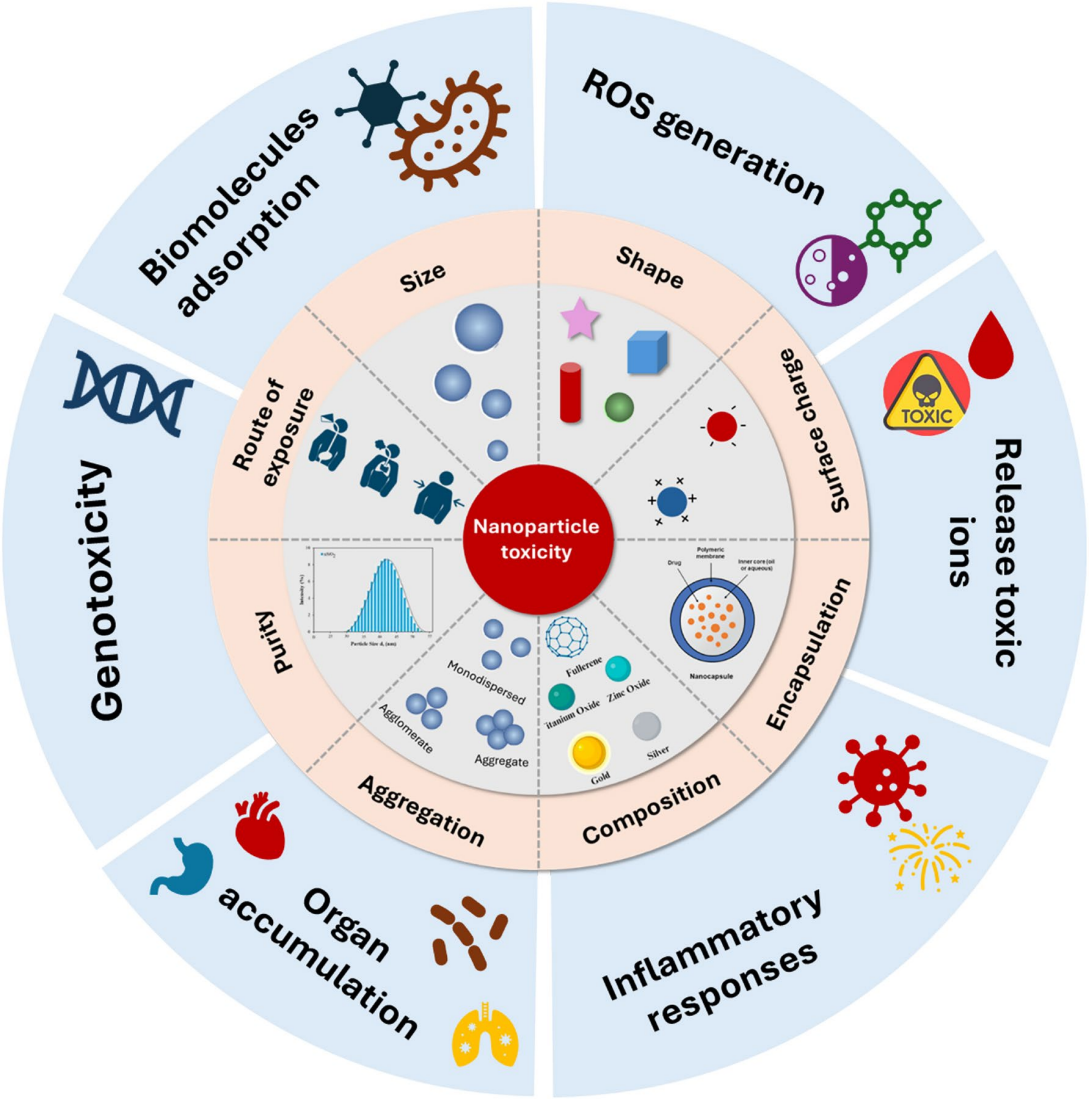
Toxicity of nanomaterials governed by an interplay composition, shape, size, surface charge, encapsulation, and route/duration of exposure and their reported toxicity mechanisms.

After converting any material into nanoparticles, a completely new substance is formed with entirely different properties. Therefore, ignoring the properties of the prepared MNM during the assessment of the environmental impact renders the assessment process unmeaningful. The toxic effects of MNMs are governed by an interplay of some factors, including composition, shape, size (dry and wet), surface charge, encapsulation, and route/duration of exposure^23^. Fig. 3 summarizes the different factors that affect the toxicity behaviors and related mechanisms of MNMs. The toxic behaviors of MNMs, based on their physicochemical properties and penalty point assignment, are presented in Table S2.

According to the OECD principles, the toxicity of MNMs is an interplay of composition, size, and shape. For the same composition, changing shape or size can significantly affect their toxicity. The reduction of particle size to the nano range improves its reactivity and surface properties. At the same time, particle size reduction reduces the protein corona formation^24^ and improves particle cell permeability, nucleus permeability, and penetration of skin barrier^22^, blood brain^25^, and reproductive organs barriers^26^. In addition, this reduction increases the reactive surface area and rate of release of toxic ions. Moreover, the hydrodynamic diameter is considered a crucial parameter affecting the environmental and biological fate of MNMs^27^. Also, the shape and aspect ratio of the nanoparticle affect their fate. The tubes and rods are reported for their high toxicity due to their high aspect ratio, hard phagocytosis, large contact surface, and sharp edges^28^.

### Exposure

The use case of the prepared MNM, for analytical, environmental, or water treatment applications, determines the degree of exposure^2^. Also, nanomaterial’s hydrophobicity increases the possibility to penetrate into cells and accumulate inside different organisms^29^.

The degree of exposure affects the toxicity of MNMs by influencing the number of nanoparticles that enter the body. Additionally, the direct addition of MNMs to water, as seen in photocatalytic decontamination processes (e.g., TiO_2_), poses significant environmental risks. These materials can persist in water and may affect various organisms through contact or ingestion. Furthermore, the transformation products generated from these processes are often unknown, and their toxicity profiles are typically not provided^30^. However, research focused on the separation, recovery, and reuse of photocatalytic materials for the detoxification of actual wastewater is insufficient.

### Life cycle analysis (LCA)

LCA is characterized as a methodical procedure that assesses the environmental dimensions and potential effects linked to a product, process, or service. LCA offers a comprehensive overview of environmental impacts and aids in identifying improvement opportunities that may not be evident when analyzing a single stage of the product’s existence^31^. The key environmental indicators of LCA, including carbon footprint, energy consumption, resource depletion, and waste disposal, are usually employed for greenness assessments^31^.

Due to their toxicity, end-of-life management is a critical aspect of the LCA of MNMs to prevent contamination and ensure that the advantages of using these advanced materials do not result in unintended ecological harm^31^. Different disposal methods are usually employed, including landfilling, incineration, and recycling. Among the disposal methods, recycling remains the greenest option, while landfilling and incineration are considered less green.

## The concept of the Nano Eco-Scale

### Ideal safe nanomaterial

The ideal safe nanomaterial can be characterized by the elimination or minimal use of hazardous reagents, minimal energy use, safety of product, and no waste generation. Unfortunately, the ideal nanomaterials are of limited occurrence even using the greenest possible choices. Some examples are the polymer and drug nanoparticles exhibiting particle size between 60 and 100 nm to improve the solubility. In the preparation of many nanoparticles, we propose that the ideal nanomaterial-based process should fulfill the following criteria:

- (1) A synthesis protocol that uses safe reagents, consumes less energy, and produces fewer impurities and emissions.
- (2) Nanomaterial that does not have a toxic effect.
- (3) A use case that does not threaten the ecosystem due to high exposure rates.
- (4) No nano-waste is produced.

### Penalty points and Nano Eco-Scale score

The environmental toxicity of MNMs arises from various properties that can be detrimental to living cells. These characteristics may negatively affect humans, microorganisms, plants, soil and water quality, air purity, agricultural productivity, pollution levels, and aquatic ecosystems. For each of the nanomaterial eco-toxicity parameters (synthesis, MNM’s safety, exposure, and waste), penalty points are assigned if it departs from the ideal safe nanomaterial (Table 1). The MNM-based analytical method is given a score of 100-penalty points and classified as excellent green/low-risk (>80), acceptable greenness/moderate-risk (60-80), poor greenness/high-risk (< 60).

**Table 1:** The penalty points (PPs) to calculate Nano Eco-Scale score.

**Process**			
			**Total PP**
**Approach**^15^	Top-down Bottom-up		0 3
**Precursors**	Edible natural Non-edible natural Low-toxicity chemical (None/Warning) Hazardous chemical (Danger)		0 1 2 3
**Solvent**	None Green Toxic/flammable/corrosive		0 1 2
**Energy consumption (CO**_**2**_** emission,**	< 0.1 0.1-1.0 >1.0		0 1 2
**Hazard**	Hermitization Emission of vapor and gasses		0 1
**Product**			
		**Subtotal PP**	**Total PP**
Particle composition *	Organic CBNMs Ceramic Low toxicity metal High toxicity metal/ carbon nanotubes	1 2 3 4 5	Subtotal PP x risk factors
		**Risk factors**	
**Size (nm)** (D: diameter HD: hydrodynamic diameter)	D >60 D 10-60/HD >100 D 10-60/ HD<100, HD not measured D <10/ HD >100 D <10/ HD <100, HD not measured	1 1.5 2 2.5 3	
**Shape**	Dots/ spheres Sheets/Plates Rods/Tubes/ Fibers/stars/cubes	1 1.5 2	
**Charge**	Negative/ surface modified Positive/not tested		0 1
**Aggregation**	Aggregates Monodispersed		0 1
**Protein corona**	Formed Not formed/ Not tested		0 1
**Cell toxicity**	Not cytotoxic Cytotoxic Genotoxic ROS generation/not tested		0 1 2 3
**Exposure**			
**Use case**	Solid sensor/ immobilization sorbent Liquid sensor Water treatment		0 1 2 3
**Nature**	Hydrophobic Hydrophilic/ Not reported		0 1
**Waste**			
**Amount**	<1 mL or g 1-10 mL or g >10 mLor g		0 1 2
**Treatment**	Reuse/ Recycle Degradation/ Restoration Passivation No treatment		0 1 2 3
**Degradability**	Biodegradable Persistent		0 1

PPs are added for each compositional element in nanoparticles or nanocomposites.

Firstly, the preparation process was evaluated in respect to the approach, reagent sustainability and safety, energy consumption, and emissions. Three penalty points were assigned to MNM prepared using the bottom-up approach. These points were based on three relevant risk factors associated with this method: the presence of high impurities (by-products and unreacted intermediates), uncertainty regarding the chemical composition, and lower production yield^15^. All the remaining parameters are organized into categories that are assigned an increasing number of penalties based on their reported toxic effects. Regarding energy consumption, in terms of CO_2_ emissions, the process was considered totally safe (zero penalty points) for CO_2_ emission less than 0.1 kg CO_2_/kWh^21^ and assigned penalty points for higher consumption (Table 1). A maximum of three penalty points were assigned for the use of hazardous chemicals (GSH: danger signal word) and three penalty points for the use of toxic solvents. Finally, the use of open vessel reactions was assigned one penalty point due to the emission of vapors and gases. The whole part is omitted during the assessment of naturally occurring nanoparticles or methods, such as those extracted from cooked food or cell cultures, or commercial products, where no synthetic procedures were performed^32,33^ .

The most effective aspect of the scale pertains to the toxicity of the MNM product. Nanoparticle composition is considered a main deferential factor for their toxic effects^34^. To address the impact of dissolution that leads to the release of toxic ions, as outlined by the OECD, metal nanoparticles are categorized into two groups: low-risk (ZrO₂, CeO₂, SnO₂, MgO, Au, TiO₂, Fe₂O₃)^35^ and high-risk (Ag, Cd, Pb, Hg, CuO, ZnO, CaO)^36,37^. The high-risk metals have the potential to dissolve, releasing toxic ions into the environment or changing the pH of the medium^38^. According to the composition, five MNM classes were presented with high-risk metal nanoparticles and carbon nanotubes (as an exception) were assigned the highest penalties (Table 1). Due to the interplay among the composition, shape, and size of nanoparticles affecting their toxicity, a risk-based approach has been proposed to calculate the total PPs. This approach is adapted from the Analytical Eco-scale, where reagent PPs were calculated based on the interaction of quantity, hazard pictograms, and the signal word^10^. The total PPs have been calculated based on the composition PPs, which are increased by a factor related to the shape and size of the nanoparticles. The shape of the nanoparticle can increase its toxic effects by escaping the immune system (making phagocytosis more difficult), resulting in tissue damage due to sharp edges and heightened surface reactivity because of a higher aspect ratio^39^. High aspect ratio nanomaterials (HARN) include structures like carbon nanotubes, nanowires, nanorods, and nanofibers that have a long, thin shape with a high length to diameter ratio. Their toxicity is driven by length, rigidity, and persistence, which can lead to frustrated phagocytosis, chronic inflammation, and asbestos-like effects in the lungs^40^. The particle size contributes to increased toxic effects due to alterations in cell penetration ability, phagocytosis, surface area, and the potential for corona formation^28^ (Table S2). For instance, the toxic effect of silver nanoparticles increases as the particle diameter is decreased. Risk factors were assigned to both particle shape (1, 1.5, and 2) and particle size (1, 1.5, 2, 2.5, and 3) (Table 1). As reported in literature, a size range between 10 and 60 nm was the most favorable hydrodynamic size for cellular uptake, regardless of NP composition and zeta potential^41^, while particles below 10 nm were more associated with genotoxicity^42^. Nanoparticles below 10 nm diameter or , 100 nm hydrodynamic diameter were assigned the highest penalties due to their large surface area, excessive ROS formation, ability to escape immunity, ability to penetrate nuclear membrane to induce genotoxic effect, and low tendency to form a protein corona^43^.

Also, an additional PP was assigned to nanoparticles with a positive surface charge due to their reported ability to disrupt cell membranes by pore formation^44–46^. On the other hand, nanoparticles with negatively charged surfaces as well as those surface-modified to mitigate their cytotoxic effects were assigned no penalty points^47^. In addition, the occurrence of nanoparticles in the form of aggregates or agglomerates not only affects their size but also their environmental fate and biological toxicity. Thus, MNMs prepared as aggregates or agglomerates are assumed to be less toxic than the monodispersed particles^2^. The biocompatibility of a biomaterial refers to its ability to effectively perform its intended function in a medical therapy without causing any undesirable local or systemic effects in the recipient. Additionally, it should promote the most suitable beneficial cellular or tissue response in that specific context while optimizing the clinically relevant performance of the therapy. For instance, silica-coated nanoparticles, superparamagnetic iron oxides, dendrimers, and mesoporous silica particles have been shown to enter cells without compromising cell survival^48^. Apart from the ideal nontoxic MNMs, cytotoxic, genotoxic materials are assigned penalty points (Table 1) considering MNMs associated with ROS generation the most toxic. Also, the protein corona formation is important to make the behavior of the MNMs predictable^49^. Accordingly, an MNM showing no corona formation is assigned one penalty. Also, to encourage thorough characterization, a default rule of assigning the highest penalty if data is absent or not tested was considered. This section is highly important as it would encourage the researchers to extensively recognize the hazardous effects of their newly prepared MNM and provide the necessary data for ecologists and nanotoxicologists to do their role in making use of these double-edged fascinating materials.

Regarding the exposure, the use case and hydrophobicity were considered. The exposure assessment is presented as a screening-level approximation intended to provide initial estimates of potential exposure, not as a comprehensive environmental risk model. The use case, solid sensor, sorbent, liquid sensor, and water treatment determine the environmental fate of the investigated MNM. The solid sensors with immobilized MNMs are considered the greenest option and assigned no penalties. The highest penalties were assigned to water treatment due to the associated risk of persistence. Also, hydrophobicity is an important factor that determines the degree of dissemination into water supplies. It profoundly influences environmental fate and transformation, how nanoparticles travel through water, soil, and air, aggregate, and bioaccumulate in ecosystems. Hydrophilic MNMs are colloidally stable, resist aggregation, favour eco-corona formation^50^. Thus, one penalty was assigned to hydrophilic MNMs.

Finally, the MNM was evaluated as a waste product, in addition to the waste generated during synthesis, in terms of, amount, treatment/disposal, and degradability (Table 1). Recovery, reuse, or recycle is considered the greenest option and assigned no penalties.

On the proposed Nano Eco-Scale, the best-case scenario results in a score of 99. The worst-case MNM-based method, which uses a single high-risk metal nanomaterial, showed a score of 43. The score could be further reduced for methods that use multiple nanomaterials or nanocomposites made from more than one type of nanomaterial.

## Examples of Nano Eco-Scale calculation

In this section, several examples of MNMs were evaluated for their greenness using the developed Nano Eco-Scale. Both the top-down and bottom-up approaches are represented in these examples. MNM for different application fields were included taking into consideration the variability in composition, shape and environmental fate.

### Carbon dots prepared using natural precursors

In this sub-section the developed Nano Eco-Scale was applied for greenness evaluation of two CBNM preparations; a naturally occurring sweet potato-derived CDs^51^ (Fig. S1A and Table 2), lab-prepared CDs from natural non-toxic precursors^52^ (Fig. S1B and Table 3), and another lab-prepared metal-CD core-shell nanoparticles^53^ (Fig. S1C and Table 4). All the tested showed green behaviour. Despite being obtained from cooked food, the sweet potato-derived CDs showed a score of 81 which is lower than expected due to incomplete characterization and improper waste disposal^51^. On the other hand, CDs synthesized from lemon juice showed moderate toxicity risk that is ascribed to incomplete characterization, improper waste disposal, and less efficient energy consumption^52^. Also, the presence of metal in the nanoparticle renders it less safe with a Nano Scale-score of 67. The presence of carbon coat might reduce MNM’s toxicity by reducing the release of toxic Ag ions^54^.

**Table 2:** Penalty points (PPs) for sweet potato derived CDs^51^ on the proposed Nano Eco-Scale.

**Product**			
		**Sub-total PP**	**Total PP**
**Particle composition**	CBNMs	2	2x3x1=6
**Size (cytotoxicity)**	D <10/ HD not measured	Risk factor 3	
**Shape**	Dots	Risk factor 1	
**Charge**	Negative		0
**Aggregation**	Monodispersed		1
**Cell toxicity**	Not tested		3
**Protein corona**	Not tested		1
**Exposure**			
**Use case**	Liquid sensor		2
**Nature**	Hydrophilic		1
**Waste**			
**Volume**	>10 mL		2
**Treatment**	No treatment		3
**Degradability**	Biodegradable		0
$\sum \mathbf{P}\mathbf{P}$			19
**Nano Eco-Scale Score**			100-19=**81**

**Table 3:** Penalty points (PPs) for lemon juice derived CDs^52^ on the proposed Nano Eco-Scale.

**Preparation**			T**otal PP**
**Approach**	Bottom-up		3
**Precursors**	Hazardous chemical (Danger)		3
**Solvent**	Green (Water)		1
**Energy consumption**	CO_2_ emission kWh per run= 3x3x0.247=2.2		2
**Hazard**	Hermitization		0
**Product**		**Sub-total PP**	
**Particle composition**	CBNM	2	2x3x1 = 6
**Size**	D <10/ HD not measured	Risk factor 3	
**Shape**	Dots/ spheres	Risk factor 1	
**Charge**	Not tested		1
**Aggregation**	Monodispersed		1
**Protein corona**	Not tested		1
**Toxicity**	Not tested		3
**Exposure**			
**Use case**	Liquid sensor		2
**Nature**	Hydrophilic		1
**Waste**			
**Amount**	> 10 mL		2
**Treatment**	No treatment		3
**Degradability**	Biodegradable		0
	$\sum \mathrm{P}\mathrm{P}$		29
	Score		100-29=71

**Table 4:** Penalty points (PPs) for Ag-CDs core-shell nanoparticles^53^ on the proposed Nano Eco-Scale.

**Preparation**			
			**Total PP**
**Approach**	Bottom-up		3
**Precursors**	Hazardous chemical (danger)		3
**Solvent**	Water		1
**Energy consumption**	0.1-1		1
**Hazard**	Emission of vapor and gasses		1
**Product**		**Sub-total PP**	
**Particle composition**	Metal	5	5x2x1 = 10
**Size**	D 10-60/ HD not measured	Risk factor 2	
**Shape**	Dots/ spheres	Risk factor 1	
**Charge**	Surface modified		0
**Protein corona**	Not tested		1
**Aggregation**	Monodispersed		1
**Toxicity**	Not tested		3
**Application**			
**Use case**	Liquid sensor		2
**Nature**	Hydrophilic		1
**Waste**			
**Amount**	> 10 mL/g		2
**Treatment**	No treatment		3
**Degradability**	Persistent		1
	$\sum \mathrm{P}\mathrm{P}$		33
	Score		100-33=67

### Nanomaterials for water treatment

A representative example for the utilization of TiO_2_ in water treatment was evaluated using the proposed Nano Eco-Scale scoring system (Fig. S1D and Table 5). Ag/MoO_3_/TiO_2_ nanocomposites were synthesized via sol-gel method. The nanocomposites were immobilized on a borosilicate glass reactor and applied for photocatalytic removal of methyl orange under UV irradiation^55^. The method showed a Nano Eco-Scale score (52) due to the synthesis approach, highly toxic metals, and water treatment application.

**Table 5:** Penalty points (PPs) for Ag/MoO_3_/TiO_2_ nanocomposites^55^ on the proposed Nano Eco-Scale.

**Preparation**			
			**Total PP**
**Approach**	Bottom-up		3
**Precursors**	Hazardous chemical (Danger)		3
**Solvent**	Water/ ethanol		1
**Energy consumption**	> 1		2
**Hazard**	Emission of vapor and gasses		1
**Product**			
**Particle composition**	Ag (5x2), Mo (4x2), Ti (4x2)		26
**Size**	D 10-60/ HD not measured	2	
**Shape**	Dots/ spheres	1	
**Charge**	Not tested		1
**Aggregation**	Aggregates		0
**Protein corona**	Not tested		1
**Safety testing**	Not tested		3
**Application**			
**Use case**	Water treatment		3
**Nature**	Hydrophilic		1
**Waste**			
**Amount**	> 10 mL		2
**Treatment**	Reuse		0
**Degradability**	Persistent		1
	$\sum \mathrm{P}\mathrm{P}$		48
	Score		100-48=52

### Nanomaterial-based sorbents

Different MNM-based sorbents were evaluated using the Nano Eco-Scale tool. These examples included calcium oxide-silica nanobeads Fig. S1E^56^ and graphene nanosheets Fig. S1F^57^, as shown in Table S3 and S4. For these sorbents, the graphene nanosheets (81) showed superior greenness over the CaO-coated silica nanobeads (71). However, the incomplete characterization of both materials resulted in score reduction.

## Case studies and tool verification

To ensure the scientific credibility of the developed tool, the Nano Eco-Scale scores were correlated with reported toxicity indicators (e.g., IC50, ROS generation) for citrate-capped silver (AgNPs)^58^ and gold nanoparticles (AuNPs)^59^. The Nano Eco-Scale scores were calculated for two creatinine-selective nanoprobes that were prepared using similar procedures and conditions. The scores were 67 and 69 for the AgNPs and AuNPs methods, respectively (Table S5). This was found consistent with the reported IC50 of 40 μg/mL (AgNPs)^20^ and 100 μg/mL (AuNPs)^60^. The reported toxicity of AgNPs is usually attributed to the release of toxic silver ions and more ROS generation^61^.

Moreover, the developed tool was further applied to a pair of electrochemical methods, with reported electrode modification using a high-risk nanomaterial, CNTs. The first method uses a modified glassy carbon electrode modified with polydopamine-multiwalled carbon nanotubes (MWCNTs) for simultaneous electrochemical determination of biocompounds in biological fluids^62^. The MWCNT method showed a score of 67 (Table S6), which could decrease to 57 upon using MWCNT with a diameter less than 10 nm. The other method uses a nanocomposite of two high-risk nanomaterials, multi-walled carbon nanotubes and cobalt oxide nanoparticles (MWCNT/Co_3_O_4_)^63^. The MWCNT/Co_3_O_4_ method showed a score of 49 (Table S7), reflecting the additive hazards due to the use of two high-risk MNMs. Using smaller diameters, the score could drop to 39 reflecting the reported higher toxic effects of MWCNTs with tiny diameters (less than 10 nm) that might create membrane instability and release of intracellular components^64^.

Furthermore, the greenness of a reported low-risk nanomaterial, chitosan nanomicelles, has been evaluated using the developed tool^65^. Acting as selective sensing probe, the fluorescence of chitosan nanomicelles was quenched and recovered when Fe^3+^ ions were combined and released from chitosan nanomicelles, respectively. The chitosan nanomicelles gave a score of 88, indicating excellent greenness (Table S8). However, the obtained score is apparently lowered due to the generation of large amounts of waste and incomplete characterization.

## Comparison with reported metric tools

The comparative analysis presented in Table 6 highlights a clear distinction between existing green chemistry metrics and nanotoxicity assessment frameworks. Conventional greenness tools, such as Eco-Scale^9^, E-factor^66^, and Analytical Eco-Scale^10^, offer simple, rapid, and quantitative evaluations, making them highly suitable for comparison of greenness. However, these approaches do not account for nano-specific properties or associated safety concerns. In contrast, nanotoxicity-oriented tools such as NanoRiskCat^67^, LICARA NanoSCAN^68^, and DF4nanoGrouping^69^ incorporate material-specific risk factors, including exposure and hazard, but require extensive datasets and specialized expertise, which can limit their applicability during early design stages. Within this context, the Nano Eco-Scale provides an integrated approach that bridges this gap by combining green chemistry principles with nano-specific considerations into a single tool. This positioning enables more informed and efficient decision-making while maintaining practical usability, although further validation and refinement remain necessary to ensure robustness across diverse nanomaterial systems. However, the Nano Eco-Scale is intended as a screening-level conceptual framework rather than a fully validated predictive model. Also, the Nano Eco-Scale framework does not adequately account for transport mechanisms, transformation products, persistence, or bioaccumulation pathways, which are essential components of realistic environmental risk assessment. In addition, significant gaps remain in understanding release probability, environmental persistence kinetics, and realistic exposure scenarios of manufactured nanomaterials, limiting accurate assessment of their environmental fate and toxicity. Moreover, it is highly recommended to establish a clear standard definition of biocompatibility along with a unified evaluation protocol tailored to nanomaterials.

**Table 6:** Comparative evaluation of green chemistry, analytical, and nanotoxicity tools versus the nano eco scale.

**Tool**	**Domain**	**Scope**	**Input Complexity**	**Output Type**	**Nano-Specific Factors**	**Environmental Coverage**	**Ease of Use**	**Ref.**
**Eco-Scale**	Green chemistry	Reaction-level	Low	Numerical score (0–100)	None	Yield, cost, safety, and conditions	High	^9^
**Analytical Eco-Scale**	Green analytical chemistry	Method-level	Low–Medium	Numerical score (0–100)	None	Reagents, energy, waste, and occupational hazards	High	^10^
**E-factor**	Green chemistry	Process-level	Low	Ratio (kg waste/kg product)	None	Waste generation only	High	^66^
**NanoRiskCat**	Nanotoxicity	Material-level	Medium	Risk categories (low/medium/high)	Yes	Exposure and hazard	Medium	^67^
**LICARA NanoSCAN**	Nano risk & sustainability	Lifecycle	High	Multi-dimensional scoring	Yes	Economic, environmental, and social	Low	^68^
**DF4nanoGrouping**	Nanotoxicity	Material grouping	High	Hazard grouping	Yes	Toxicity focus	Low	^69^
**Nano Eco Scale**	Integrated green analytical chemistry and nanotoxicity	Method level	Low–Medium	Numerical score (0–100)	Yes	Yield, waste, energy, and nanotoxicity	High	This work

Also, the Nano Eco-Scale tool is mainly focused on analytical and environmental applications. Nevertheless, the scale might be adapted in the future for MNMs intended for biomedical use by incorporating parameters such as degradation kinetics, biopersistence, dissolution in biological fluids, route of administration, and elimination profile. Moreover, comprehensive statistical validation and benchmarking would require standardized datasets that remain limited in the current nanotoxicology literature and therefore represent important directions for future work. Also, the current multiplicative formulation should therefore be interpreted as a screening-level conceptual approach rather than a definitive predictive mathematical model, while alternative weighting and scoring strategies may be explored in future studies as more standardized datasets become available. Future work should include formal parameter sensitivity and uncertainty assessment approaches as more standardized datasets become available.

## Conclusion

In this work, a new metric tool, Nano Eco-Scale score, was introduced to evaluate the greenness of nanomaterials-based analytical methods, across synthesis, product, exposure, and safety. The tool framework is an integration of green analytical chemistry principles, REACH regulations, and OECD principles for safe use of manufactured nanomaterials (MNMs). The scoring system is based on penalty points assigned for deviations from ideal green nanomaterial and expressed as a final score of 100 minus the total penalty points. The higher the score, the less the ecotoxic effect of the MNM-based applications. The toxicity behavior of MNMs was evaluated based on the key characteristics including composition, particle size, shape, surface charge, and exposure conditions, which collectively influence their interaction with biological and environmental systems. The approach could enable the comparison between MNM-methods based on environmental performance and potential hazard. The tool also highlights the importance of detailed MNM characterization and the need to account for toxicity effects that are often overlooked, particularly in analytical and industrial applications. The Nano Eco-Scale tool is not intended for the evaluation of nanotoxicity of new materials nor decision making regarding their clinical use or safety prediction. It is simply used for comparing the potential environmental risks associated with the new MNM-based analytical and environmental methods. An approach that supports responsible development and use of nanotechnology, ensuring that functional benefits are achieved without compromising human health or environmental integrity. Also, the proposed tool may also help facilitate systematic data collection and comparative evaluation that could support future refinement, calibration, and classification approaches.

## Supplementary Information

Supplementary Information.

## Data Availability

All data generated or analyzed during this study are included in this published article and its additional file.
