# Supplementary material for: Nano Eco-Scale: A scoring system for the assessment of the greenness and safety of manufactured nanomaterials for analytical and environmental applications
Source: Sci Rep. 2026 Jul 21;16:22810. doi: 10.1038/s41598-026-62228-5 (PMC13389090; doi:10.1038/s41598-026-62228-5)
Supplement: Supplementary file 1 — Supplementary Information. [file 41598_2026_62228_MOESM1_ESM.docx]

**Supplementary**

**Nano Eco-Scale: A scoring system for the assessment of the greenness and safety of manufactured nanomaterials for analytical and environmental applications**

Aya A. Abdella

*Department of Pharmaceutical Analytical Chemistry, Faculty of Pharmacy, Tanta University, Egypt.*

*Corresponding author e-mail: [**Aya.atef.86@pharm.tanta.edu.eg**](mailto:Aya.atef.86@pharm.tanta.edu.eg), [**Aya.atef.86@gmail.com**](mailto:Aya.atef.86@gmail.com)

Telephone number: +2/01554052033

Fax number: +2/0403335466

**
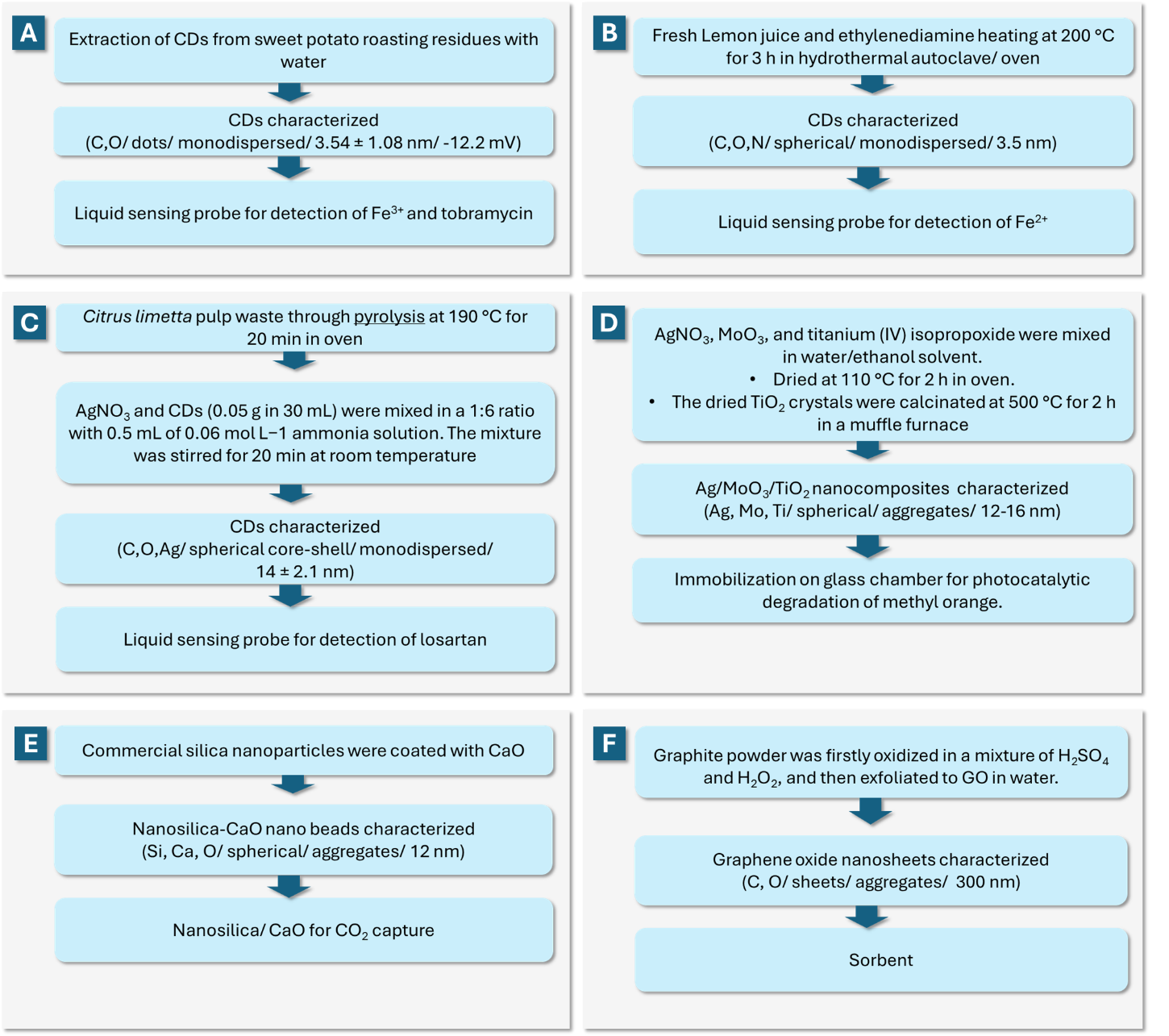
**

**Fig. S1.** Procedure and characterization parameters of (A) sweet potato derived carbon dots for spectrofluorimetric determination of Fe^3+^ and tobramycin ^51^, (B) CDs from lemon juice for spectrofluorimetric determination of Fe^2+ 52^, (C) Ag/CDs core-shell nanoparticles for spectrofluorimetric determination of losartan ^53^, (D) Ag/MoO_3_/TiO_2_ nanocomposites for photocatalytic degradation of methyl orange ^55^, (E) nanosilica-CaO nanoparticles for CO_2_ capture^56^, and (F) graphene oxide nanosheets ^57^.

**Table S1.** Power of different instrumentations employed in MNM preparation for the calculation of carbon footprint.

|  |  | **Power (kW)** |
| --- | --- | --- |
| **Heating** | Gas oven  Gas stove | 1.5-2.0  2.0-3.0 |
|  | Hot plate | 1.0-1.5 |
|  | Lab oven | 1.5-3.0 |
|  | Lab furnace | 1.0-5.0 |
|  | Muffle furnace | 2.0-10.0 |
| **Size reduction** | Mechanical milling or ball milling | 0.25 -10.0 |
|  | Thermal evaporation system | 0.5-5.0 |
|  |  |  |

**Table S2.** Classification of MNMs according to their reported toxic behaviour and toxicity levels

| Nanomaterial parameter |  | Toxicity level | Related toxic behaviour | Penalty points | Ref |
| --- | --- | --- | --- | --- | --- |
| Interplay of Composition, size, and shape (Subtotal penalty points x risk factors) | | | | | |
| Composition  (subtotal penalty points) | Organic | Less toxic than other NPs | Relatively safe | 1 | ^29^ |
|  | CBNMs | Least toxicity | Different toxic effects based on size, charge and surface functionalization. | 2 | ^30^ |
|  | Silica | High toxicity | Cytotoxicity/ Lung toxicity (inhalation) | 3 | ^31^ |
|  | Low toxicity metals  ZrO₂/ CeO₂/ SnO₂/ MgO/ Au/ TiO_2/_ Fe_2_O_3_ | Higher than ceramics | Cytotoxicity  Lung toxicity (inhalation)  Genotoxicity  ROS generation  Low water solubility/ less ion release | 4 | ^32^  ^33^  ^34^ |
|  | High toxicity metals  Ag/ Cd/ Pb/ Hg/ CuO/ ZnO  Carbon nanotubes | High toxicity | Cytotoxicity/ Lung toxicity (inhalation)  Lung toxicity (inhalation)  Genotoxicity  ROS generation  Dissolve/ toxic ion release | 5 | ^35^ |
| Shape  (Increases the penalty points by a risk factor) | Dots | Less toxic | Low mechanical damage/ low aspect ratio/ easier phagocytosis | Risk factor=1 | ^30^ |
|  | Sheets | Moderately toxic | Sharp edges/ contact larger surface | Risk factor=1.5 | ^36^ |
|  | Tubes/fibres | Highly toxic | Sharp edges/ high aspect ratio/ large contact surface/ persist in tissues due to difficult phagocytosis | Risk factor=2 | ^28^ |
| Size  (Increases the penalty points by a risk factor) | D >60 | Less toxic | Detected by immune system/ less penetration/hard corona formation | Risk factor=1 | ^37^  ^27^ |
|  | D 10-60/HD >100 | Intermediate | Moderate penetration | Risk factor=1.5 |  |
|  | D 10-60/ HD<100, HD not measured | Intermediate | Moderate penetration | Risk factor=2 |  |
|  | D <10/ HD >100 | Intermediate | Moderate penetration/ less biological toxicity | Risk factor=2.5 |  |
|  | D <10/ HD <100, HD not measured | Highly toxic | Genotoxic/less corona formation/ escape immunity/ penetrate nuclear membrane | Risk factor=3 |  |
| Charge | Positive | More toxic | Penetrate cell | 0 | ^37^ |
|  | Negative | Less toxic | Less cell penetration | 1 |  |
| Aggregation | Aggregates | Less toxic | Smaller active surface area and contact surface/ immune detectability | 0 | ^24^ |
|  | Monodispersed | More toxic | Larger active surface area and contact surface/ less immune detectability | 1 |  |
| Protein corona | Formed | Less toxic | Expected behaviour/ less release of toxic ions and ROSs | 0 | ^24^ |
|  | Not formed/ Not tested | More toxic | Unexpected behaviour/ more release of toxic ions and ROSs | 1 |  |
| \|  \| \| --- \|   Toxicity | Not cytotoxic  Cytotoxic  Genotoxic  ROS generation/not tested | | More penalty points for more toxic or untested MNM. | 0  1  2  3 | ^24^ |

**Table S3.** The penalty points (PPs) for nanosilica-CaO nanoparticles ^56^ on the proposed Nano Eco-Scale

| Product | | | |
| --- | --- | --- | --- |
|  |  |  | **Total PP** |
| Particle composition | Silica (3x2) CaO (5x2) | | 16 |
| Size | D 10-60/ HD not measured | Risk factor 2 |  |
| Shape | Dots/ spheres | Risk factor 1 |  |
| Charge | Not tested |  | 1 |
| Protein corona | Not tested |  | 1 |
| Aggregation | Aggregates |  | 0 |
| Toxicity | Not tested |  | 3 |
| Exposure | | | |
| Use case | Solid sorbent | | 1 |
| Nature | Hydrophilic | | 1 |
| Waste | | | |
| Amount | >10 g | | 2 |
| Treatment | No treatment | | 3 |
| Degradability | Persistent | | 1 |
| $\sum\mathbf{PP}$ | | | 29 |
| Score | | | 100-29=71 |

**Table S4.** The penalty points (PPs) for green graphene oxide nanosheets ^57^ on the proposed Nano Eco-Scale

| Preparation |  |  |  |
| --- | --- | --- | --- |
|  |  |  | **total PP** |
| Approach | Top-down | | 0 |
| Precursors | Hazardous chemical (Danger) | | 3 |
| Solvent | Water | | 1 |
| Energy consumption | < 0.1 | | 0 |
| Hazard | Emission of vapor and gasses | | 1 |
| Product |  |  |  |
|  |  | **Sub-total PP** | **total PP** |
| Particle composition | CBNM | 2 | 2x1x1.5  = 3 |
| Size | > 60 | Risk factor 1 |  |
| Shape | Sheets | Risk factor 1.5 |  |
| Charge | Negative |  | 0 |
| Aggregation | Aggregates |  | 0 |
| Protein corona | Not tested |  | 1 |
| Toxicity Toxicity | Not tested |  | 3 |
| Application |  |  |  |
| Solid | Solid | | 1 |
| Nature | Hydrophilic | | 1 |
| Waste |  |  |  |
| Amount | > 10 mL | | 2 |
| Treatment | No treatment | | 3 |
| Degradability | Biodegradable | | 0 |
|  | $\sum\mathrm{PP}$ | | 19 |
|  | Score | | 100-19=81 |

**Table S5.** Case study: comparing citrate capped AgNPs and AuNPs using the developed Nano Eco-Scale tool

| AuNPs ^59^ | | | | AgNPs ^58^ | | |
| --- | --- | --- | --- | --- | --- | --- |
| Preparation |  |  | **Total PP** |  |  | **Total PP** |
| Approach | Bottom-up | | 3 |  | Bottom-up | 3 |
| Precursors | Hazardous chemical (danger) | | 3 |  | Hazardous chemical (danger) | 3 |
| Solvent | Water | | 1 |  | Water | 1 |
| Energy consumption | 0.1-1 | | 1 |  | 0.1-1 | 1 |
| Hazard | Emission of vapor and gasses | | 1 |  | Emission of vapor and gasses | 1 |
| Product |  | **Sub-total PP** |  |  | **Sub-total PP** |  |
| Particle composition | Metal | 4 | 4x2x1  = 8 | Metal | 5 | 5x2x1  = 10 |
| Size | D 10-60/ HD not measured | Risk factor 2 |  | D 10-60/ HD not measured | Risk factor 2 |  |
| Shape | Dots/ spheres | Risk factor 1 |  | Dots/ spheres | Risk factor 1 |  |
| Charge | Surface modified |  | 0 | Surface modified |  | 0 |
| Protein corona | Not tested |  | 1 | Not tested |  | 1 |
| Aggregation | Monodispersed |  | 1 | Monodispersed |  | 1 |
| Toxicity | Not tested |  | 3 | Not tested |  | 3 |
| Application |  |  |  |  |  |  |
| Use case | Liquid sensor | | 2 |  | Liquid sensor | 2 |
| Nature | Hydrophilic | | 1 |  | Hydrophilic | 1 |
| Waste |  |  |  |  |  |  |
| Amount | > 10 mL/g | | 2 |  | > 10 mL/g | 2 |
| Treatment | No treatment | | 3 |  | No treatment | 3 |
| Degradability | Persistent | | 1 |  | Persistent | 1 |
|  | $\sum\mathrm{PP}$ | | 31 |  | $\sum\mathrm{PP}$ | 33 |
|  | Score | | 100-31=69 |  | Score | 100-33=67 |

**Table S6.** The penalty points (PPs) for MWCNT ^62^ on the developed Nano Eco-Scale

| Preparation |  |  | total PP |
| --- | --- | --- | --- |
| Approach | Top-down |  | 0 |
| Precursors | Hazardous chemical (Danger) |  | 3 |
| Solvent | Water |  | 1 |
| Energy consumption | < 0.1 |  | 0 |
| Hazard | Emission of vapor and gasses |  | 1 |
| Product |  |  |  |
|  |  | **Sub-total PP** | **total PP** |
| Particle composition | CNTs | 5 | 5x2x2  = 20 |
| Size | D 10-60/ HD not measured | Risk factor 2 |  |
| Shape | Tubes | Risk factor 2 |  |
| Charge | Not measured |  | 1 |
| Aggregation | Aggregates |  | 0 |
| Protein corona | Not tested |  | 1 |
| Toxicity Toxicity | Not tested |  | 3 |
| Application |  |  |  |
| Solid | Immobilized | | 0 |
| Nature | Not reported | | 1 |
| Waste |  |  |  |
| Amount | > 10 mL | | 2 |
| Treatment | Reuse | | 0 |
| Degradability | Biodegradable | | 0 |
|  | $\sum\mathrm{PP}$ | | 33 |
|  | Score | | 100-33=67 |

**Table S7.** The penalty points (PPs) for MWCNT/Co_3_O_4_ ^63^ on the developed Nano Eco-Scale

| Preparation |  |  | total PP |
| --- | --- | --- | --- |
| Approach | Co_3_O_4_ (bottom-up) |  | 3 |
| Precursors | Hazardous chemical (Danger) |  | 3 |
| Solvent | Water |  | 1 |
| Energy consumption | < 0.1 |  | 0 |
| Hazard | Emission of vapor and gasses |  | 1 |
| Product |  |  |  |
|  | **Sub-total PP** |  | **total PP** |
| Particle composition | CNTs (5x2x2) Co_3_O_4_ (5x3x1) | | 35 |
| Charge | Not measured |  | 1 |
| Aggregation | Aggregates |  | 0 |
| Protein corona | Not tested |  | 1 |
| Toxicity Toxicity | Not tested |  | 3 |
| Application |  |  |  |
| Solid | Immobilized | | 0 |
| Nature | Not reported | | 1 |
| Waste |  |  |  |
| Amount | > 10 mL | | 2 |
| Treatment | Reuse | | 0 |
| Degradability | Biodegradable | | 0 |
|  | $\sum\mathrm{PP}$ | | 51 |
|  | Score | | 100-51=49 |

**Table S8.** The penalty points (PPs) for a reported low-risk chitosan nano micelles ^65^ on the developed Nano Eco-Scale

| Product |  |  |  |
| --- | --- | --- | --- |
|  |  | **Sub-total PP** | **total PP** |
| Particle composition | Organic | 1 | 1x1x1  = 1 |
| Size | > 60 | Risk factor 1 |  |
| Shape | spheres | Risk factor 1 |  |
| Charge | Not tested |  | 1 |
| Aggregation | Monodispersed |  | 1 |
| Protein corona | Not tested |  | 1 |
| Toxicity Toxicity | Not tested |  | 3 |
| Application |  |  |  |
| Solid | Liquid sensor | | 2 |
| Nature | Hydrophilic | | 1 |
| Waste |  |  |  |
| Amount | > 10 mL | | 2 |
| Treatment | Reuse | | 0 |
| Degradability | Biodegradable | | 0 |
|  | $\sum\mathrm{PP}$ | | 12 |
|  | Score | | 100-12=88 |
